# Report of a Case of Diphtheria, Followed by Epilepsy

**Published:** 1870-04

**Authors:** N. Bridge

**Affiliations:** Chicago


ARTICLE VII.—*Report of a case of Diphtheria, followed by Epilepsy.* By N. BRIDGE, M.D., Chicago.

MR. T. B. W. S., English, aged about 35 years, has been many years in America and been employed as a clerk writing most of the time. He is slim and spare of frame, but says he has always had excellent health. During the past year, as his wife since avers, reverses of fortune have made him low spirited and hypochondriacal; has had from time to time momentary attacks of mental wandering, would often stop what he was doing, look vacantly a moment, make some strange remark and then appear natural; he has seemed, at times, almost stupid, would sleep much of the time and was easily imposed upon. He came to Chicago in October, 1869, and went to work in a packing-house as a laborer.

He was attacked with diphtheria about December 1. I was called December 5. His attack did not seem severe; yet he was apponic — speaking only in a whisper, had a not very thickly-coated tongue which was somewhat dark colored. False membrane was distinct and was beginning to loosen; there was anoresid, soreness of outside of throat with slight swelling, pain, not very severe, on right side of head; had not taken to his bed, and his nights had been quite sleepless, pulse 80.

Ordered gargle potass chloras, 3 minims hydrochl. acid and 10 m. tr. belladonna every three hours. Dov. pul. 4 grs. at night. Water compresses to the throat, and nourishment.

This treatment was continued, the patient improving, till the 10th, when 2 grs. quinine was ordered every morning.

He improved steadily, his appetite gaining until the 13th when, at night eating largely of oysters, he was attacked soon after supper with vomiting and a true epileptiform convulsion; the fit was repeated several times during the early part of the night and again near midnight. Gave dose — about 20 grs. — of bromide potass., a goblet of wine, 3 grs. quinine and $\frac{1}{4}$ gr. of morph. during this time. He fell asleep at 2 o'clock in the morning and had no more trouble.

He had no more fits until the night of the 16th, taking his two grs. quinine and a little wine at meal time. He was on the 15th much excited all day, and the following day. On the 16th late

P.M., he got very indignant about something and in the early evening had another convulsion. These continued to occur every 20 or 30 minutes for some hours. The paroxysms were short, would often last not more than half a minute and seldom over a minute; they began with a sighing sound often, paleness, tonic spasm, dilation of pupils, followed by slight clonic action, flushed face, etc., as in ordinary epilepsy. He was, of course, unconscious during the fits, but was fully aware after each that it had occurred. As his face began to be flushed the pupils would contract promptly and naturally. Once in a while no muscular spasm could be detected whatever. He was covered with a cold, clammy sweat, and said he felt a terrible weakness all through him.

After the first fit the pulse went down to about 33, the respiration being faster than normal. During the fits — or that part characterized by paleness, *his heart would stop beating*, and as the face became red at the close it would begin again, but much more rapidly, beating faster than normally, but soon settling down to its former slowness. The pulse felt labored and not strong, while the first sound of the heart was short and somewhat jerking; no other abnormality could be detected in it from first to last.

At 7 P.M. he was given 3 grs. quinine, ½ gr. morph. and 4 ℥ port wine, with mustard to the chest.

Convulsions grew less and I left him quiet and more easy at 9 o'clock. At this time the pulse was 33, full but not strong; respiration about 25 — conscious and sane.

At 11½ P.M. was called to him again and asked Dr. Walter Hay to see the case with me. The paroxysms were found occurring every ten or fifteen minutes and very brief; pulse 28; weaker respiration 28; great feeling of weakness; cold, and moist skin. He was ordered whisky ℥ ss every 2 hours, with beef tea intervening; also,

| | | |
|---|---|---|
| ℞ | Ferri Bromid, - - - - - - - | ℥ j; |
| | Syr. Simp., Aqua, - . - - - | aa ℥ ij; |

Ms. teaspoonful every 4 hours, much diluted.

On the morning of the 17th he was much the same; the nurse said he got about an hour's sleep and freedom from paroxysms just before daylight. He was given 15 grs. brom. pot. and 3 grs. quinine. The paroxysms continued during the day at short intervals, and during most of the following night, not more than two hours of sleep being procured.

On the 18th — A.M. — the pulse was a little stronger and 30 per minute ; respiration 28. He had, at about daybreak, vomited a little black matter. Dil. hydrocyan. acid was given twice during the day in 2-drop doses, and for thirst, which was annoying, wine and water.

At night he felt a little better, the fits which had persisted during the day, having decreased in frequency to once in every half hour ; the pulse was 30, and weaker, and the respiration 25.

The brom. tarri. was now omitted and 15 grs. brom. pot. and gtt. 20 tr. digital. given every 4 hours alternating.

The 19th found him much weaker ; pulse and respiration the same in frequency as the day before, and the pulse was steadier. He had, during the night, gained a little sleep, and the fits were fewer. During the early part of the day the nurse blundered and gave two doses of the digitalis, double what was ordered. The general weakness increased ; the heart stopped during the most of each fit and it seemed hard to begin again, when it did it was with a nervous, jerking and weak motion. The digitalis was stopped wholly ; the other treatment continued. At 5 P.M. the pulse was 30 and a deathly coldness possessed the body ; he was at this time, and had been between all the paroxysms, perfectly sane and able to converse intelligently.

He died at 6½ P.M. in a fit.

No *post-mortem* examination was had.

This man doubtless had the beginning of epilepsy a year before his death, and would have died of it sooner or later had he escaped the diphtheria. The paroxysms he had were plainly of an epileptiform character ; nearly all their symptoms were characteristic of that disease.

Dr. Hay at once, on seeing him, expressed the opinion that there must be a mental element as a cause of the trouble, and one which reached back many months ; although the patient had assured me of his perfect health up to the time of his attack, it was learned afterward that this prediction — as recorded — was well founded.

One symptom, certainly unusual, was that of the cessation of the heart's action during the convulsions. No man or book I have consulted tells me that the heart ever stops in the fits of epilepsy; most works say nothing about it, or only by implication say it does not. This symptom was constant during the disease, occurring to a greater or less degree in every fit I observed; then the heart was weak and labored hard.

Not less unusual was the rapid breathing; he breathed, at times, as often as his heart beat, and for nearly three days the frequency of the respiration was fully one-half greater than normal. There was no disease of the lung, the air reached to every part, the sound was normal and no obstruction or feeling of suffocation existed at any time.

What is the explanation of these symptoms? What connection, if any, had they with the diphtheria?

Did the sudden development of this phase of the epilepsy occur simply as a result of debility and impoverished blood, or was it determined by the specific nature of the disease that preceded it? If the former, why did the heart stop in every fit, why the respiration so rapid? These symptoms were too constant to be accidental, they must have had a cause, what was it?

If, from the involvement in epilepsy of a part of the nervous mass near the origin of the par vagum, the function of that nerve was disturbed, why did it not affect the heart and lungs alike? Why make the action of one much more rapid than normal and that of the other less than half its wonted frequency?

Are the origins of the respective filaments of this nerve that go to the heart and the lungs so distant and distinct that they could have been so differently affected by a lesion in their neighborhood? If such a condition were supposable, is the lesion of the medulla and cord in epilepsy likely ever to be sufficiently circumscribed to allow of this?

Now, diphtheria is frequently attended with paralysis of certain muscles as sequels, occurring some days after the force of the disease is spent; while the paralysis, as a rule, is confined to a few parts, many different muscles are from time to time affected; those of the voice, the face, of respiration, and once in a while those of the heart, are involved. The paralysis is not always, if indeed often, complete, the muscle being simply impaired in power, not wholly disabled.

Is it unreasonable to believe that in this case the muscular structure of the heart was so affected; not paralyzed, but so weakened that it with difficulty moved; so much debilitated that, at the beginning of each paroxysm, by the peculiar nervous spasm or a peculiar state of the capillary circulation at that moment, it was stopped altogether, to begin again when the capillary circulation and normal nervous conditions were restored?

If such a view is admissible, may not the rapid breathing be explained by supposing that the lungs in answer to the normal action of the pneumogastric took on an increased action to more fully ærate the blood, a work which was highly necessary from the slowness with which it moved through the lungs? The character of the breathing differed in no wise from that which often occurs from the action of too much carbon, or other effete matter in the blood as in violent exercise or in a bad atmosphere. Moreover, if this notion be correct, if the walls of the heart were weakened, how did the case differ, so far as the heart was concerned, from some cases of angina pectoris, when, as authors aver, the heart dilates without power to contract and expel its contents and the patient keeps motionless for fear of death?

